# Lipoprotein Lipase (LPL) Polymorphism and the Risk of Coronary Artery Disease: A Meta-Analysis

**DOI:** 10.3390/ijerph14010084

**Published:** 2017-01-16

**Authors:** Li Xie, You-Mei Li

**Affiliations:** Department of Cardiovascular Medicine, Xinqiao Hospital, Third Military Medical University, Chongqing 400037, China; ym_ll0909@sohu.com

**Keywords:** coronary artery disease, lipoprotein lipase, polymorphism, meta-analysis

## Abstract

Background: In recent years, the lipoprotein lipase (LPL) polymorphism has been extensively investigated as a potential risk factor for coronary artery disease (CAD). However, the results of these studies have been inconsistent. Therefore, we performed this meta-analysis to explore the association between LPL polymorphism and CAD risk. Methods: The literature was searched from electronic databases such as Embase, China Biological Medicine Database, PubMed, Knowledge Infrastructure, and China National Web of Science by the key words “coronary artery disease”, “lipoprotein lipase” and “polymorphism”. All of the studies included in this manuscript met the inclusion and exclusion criteria. An odds ratio (OR) analysis using a 95% confidence interval (CI) was employed to assess the association of the LPL polymorphism with CAD susceptibility. Results: We performed a meta-analysis of 14 case-control studies including HindIII, Ser447X and PvuII polymorphism. A statistically significant increase in the risk of CAD was associated with LPL HindIII polymorphism. This included HindIII H^+^H^+^ genotype (OR = 1.28, 95% CI = 1.09–1.49, *p* = 0.002, I^2^ = 43%) and H^+^ allele genotype (OR = 1.27, 95% CI = 1.03–1.58, *p* = 0.03, I^2^ = 67%). Ser447X XX genotype (OR = 2.37, 95% CI = 1.33–4.24, *p* = 0.004, I^2^ = 53%) was also associated with CAD risk. However, PvuII polymorphism was found to have no significant association with CAD risk. Conclusions: LPL HindIII polymorphism was significantly associated with the risk of CAD. For Ser447X polymorphism, it was found that only XX genotype was significantly associated with CAD risk. Furthermore, PvuII polymorphism had no significant association with CAD risk. It was considered that LPL HindIII polymorphism might serve as a potential biomarker for CAD risk.

## 1. Introduction

Coronary artery disease (CAD) is one of the greatest causes of morbidity and mortality throughout the world. It accounts for roughly one-half of all cardiovascular deaths [1,2]. Although tobacco use and lipid metabolism disorder are the classical risk factors for CAD, some studies have found that CAD shows strong familial aggregation, and a gene variant is also a key risk marker for CAD patients [3,4]. For example, Bai et al. found that the monocyte chemoattractant protein-1 (MCP-1)-2518A>G polymorphism was associated with susceptibility to CAD, especially in Caucasians [5]. By contrast, Zhang et al. found that the COX-2–765G>C (rs20417) polymorphism was protective against CAD [6]. Meanwhile, a few studies have reported that lipoprotein lipase (LPL) gene polymorphism may be associated with CAD risk.

LPL is a key enzyme for lipid metabolism. It can hydrolyze the triglycerides of chylomicrons and very-low-density lipoprotein (VLDL) to provide free fatty acids for oxidation and utilization in the heart and other tissues, as well as for storage in adipose tissue [7,8]. The abnormal expression of LPL is part of some pathophysiological processes, such as diabetes, chylomicronemia, obesity, and atherosclerosis [9]. The LPL gene is located on chromosome 8p22. Over 100 mutations have been found in this gene [10,11]. A few studies have reported that the polymorphisms of HindIII, Ser447X and PvuII were associated with the risk of CAD [10,12,13,14]. However, these results were also controversial [14,15,16,17]. Some studies found no association between these gene variants of LPL and the risk of CAD [14,15].

In this study, we performed the meta-analysis to explore these inconsistencies between LPL gene variants and the risk of CAD. The results confirmed that the LPL HindIII polymorphism is significantly associated with increased risk of CAD. This polymorphism might serve as a potential biomarker for CAD risk. 

## 2. Materials and Methods

### 2.1. Literature Sources and Search Strategies

The literature included in this study was searched from electronic databases including Embase, China Biological Medicine Database, PubMed, Knowledge Infrastructure, and China National Web of Science. The key words were as follow: “coronary artery disease”, “lipoprotein lipase” and “polymorphism”. The relative of the references from the retrieved studies were also included. The last search was updated on 15 December 2015, with publication years ranging from 2000 to 2015.

### 2.2. Inclusion and Exclusion Criteria

The studies were selected based on the following inclusion criteria: the case-control studies discussed the polymorphism of LPL and the risk of CAD; the studies supplied the number of individual genotypes for the LPL polymorphisms in the CAD cases and controls; and the studies conform to the Hardy–Weinberg equilibrium (HWE). In addition, the following exclusion criteria were used: the study did not provide the detailed data that are typically presented in abstracts, meeting reports and reviews; the genotype frequency was not reported; and the studies repeated or overlapped other publications.

### 2.3. Data Extraction

The information was drawn out according to a standard protocol. Repeated publications were only adopted once and studies violating the inclusion criteria or providing insufficient data were excluded. The extracted data in Table 1 comprise the following items: the first author’s name, publication date, region of study, ethnicity of the sample population, number of genotypes, total number of cases and controls and HWE. Two authors (Li Xie and You-Mei Li) independently extracted the above information and disagreement was discussed by all authors to obtain consensus. 

### 2.4. Statistical Analyses

Deviation from the Hardy–Weinberg equilibrium was examined by the Chi-square test. The odds ratio (OR) and the corresponding 95% confidence interval (CI) between the case and control groups was used to assess the strength of the association between the LPL polymorphisms and the risk of CAD. For the HindIII, Ser447X and PvuII polymorphisms, we pooled the OR by comparing the risk allele carrier genotypes versus the wild-type genotype for low frequency of the homozygous variant. The statistical significance of the summary OR was determined by the Z test with the significance set as *p* value less than 0.05. If there was heterogeneity among the individual studies, the random effects model was adopted to calculate the overall OR value [18]. Otherwise, the fixed effects model was adopted [19]. To determine the publication bias, the funnel plot was used. The Egger’s linear regression test on the natural logarithm scale of the OR was used to assess the funnel plot asymmetry; the significance was set at the *p* < 0.05 level [20,21]. All statistical analyses were performed with RevMan V.5.0 software (Nordic Cochran Centre: Copenhagen, Denmark).

## 3. Results

### 3.1. Study Characteristics

**Figure 1:**
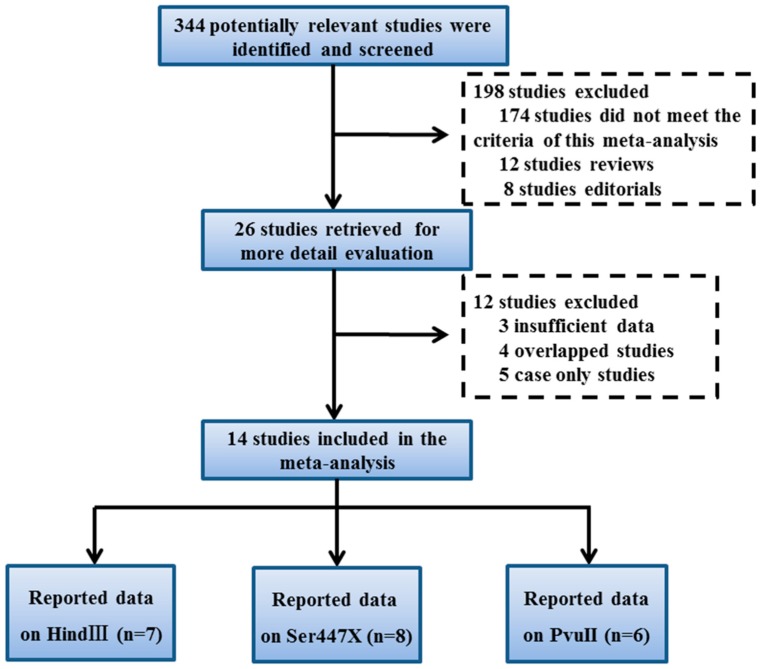
Flow of study identification, inclusion, and exclusion.

The databases of Embase, China Biological Medicine Database, PubMed, Knowledge Infrastructure, and China National Web of Science were used to identify literature for inclusion in this study. The following key words were entered: “coronary artery disease”, “lipoprotein lipase” and “polymorphism”. Originally, 224 potentially relevant studies were identified and screened. Then, these studies were carefully analyzed and 14 case-control studies were selected [13,14,15,16,17,22,23,24,25,26,27,28,29,30]. The detailed study selection process is illustrated in Figure 1. For the HindIII polymorphism and the risk of CAD, seven studies that involved 1853 cases and 1171 controls were available [13,14,15,22,25,28,30]. For the Ser447X polymorphism, eight studies that included 1519 cases and 824 controls were available [14,15,23,24,25,26,28,29]. For the PvuII polymorphism, six studies with 1064 cases and 832 controls were selected [14,15,16,17,25,27]. All of the studies included in the meta-analysis were consistent with HWE in the control populations. The detailed characteristics of the studies included in this meta-analysis are shown in Table 1. The distribution of HindIII, Ser447X and PvuII polymorphism genotype is listed in Table 2.

### 3.2. Association of the HindIII Polymorphism with CAD

**Figure 2:**
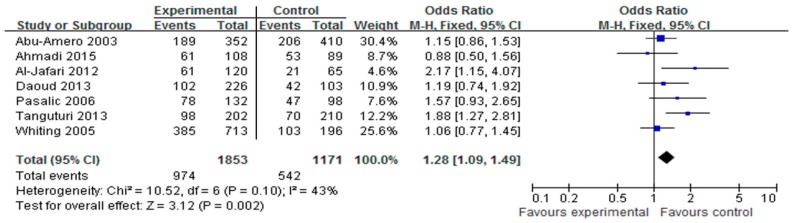
Forest plot of HindIII H^+^H^+^ genotype associated with CAD risk.

**Figure 3:**
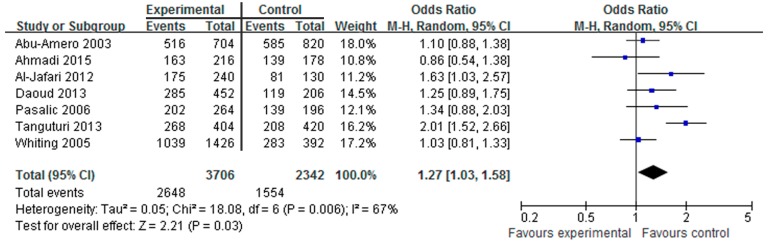
Forest plot of HindIII H^+^ allele genotype associated with CAD risk.

Data from seven studies that included 1853 cases and 1171 controls were pooled together for analysis of the association between the HindIII polymorphism and the risk of CAD. The overall data showed that individuals who carried the HindIII H^+^H^+^ genotype had a significantly increased CAD risk compared with those who carried the HindIII H^+^H^−^ and H^−^H^−^ genotypes in all subjects (OR = 1.28, 95% CI = 1.09–1.49, *p* = 0.002, I^2^ = 43%, Figure 2). Because the heterogeneity among the studies was not significant (all *p* > 0.05), the fixed-effects model was conducted. In addition, we found that the H^+^ allele genotype was significantly associated with increased CAD risk when compared with the H^−^ allele genotype (OR = 1.27, 95% CI = 1.03–1.58, *p* = 0.03, I^2^ = 67%, Figure 3). It was also determined that the heterozygous and recessive genetic models had no association with the risk of CAD.

### 3.3. Association of the Ser447X Polymorphism with CAD

**Figure 4:**
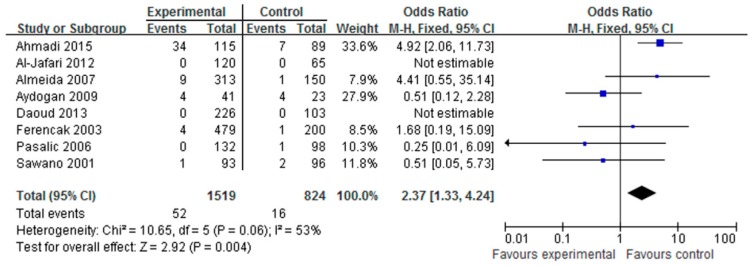
Forest plot of Ser447X XX genotype associated with CAD risk.

Eight studies examining CAD were included for the evaluation of the association with the LPL Ser447X polymorphism. As the heterogeneity was not significant (*p* = 0.06), the fixed-effects model was used. It was determined that the individuals who carried the Ser447X XX genotype had a significantly increased CAD risk compared with those who carried the Ser447X other genotypes in all subjects (OR = 2.37, 95% CI = 1.33–4.24, *p* = 0.004, I^2^ = 53%, Figure 4). In the X allele genetic model, the heterogeneity was significant (*p* < 0.0001), and the random-effects model was used for analysis. It was found that X allele genetic model had no significant association with CAD risk (OR = 1.04, 95% CI = 0.60–1.80, *p* = 0.90, I^2^ = 87%). In addition, we analyzed other genetic models and found that the heterozygous and dominant genetic models had no association with the risk of CAD.

### 3.4. Association of the PvuII Polymorphism with CAD

Data from six studies that included 1064 cases and 832 controls were pooled together to analyze the association between the PvuII polymorphism and the risk of CAD. For the dominant genetic model, the fixed-effects model was conducted because the heterogeneity was not significant (*p* = 0.45). It was determined that this genetic model had no significant association with the risk of CAD (OR = 0.96, 95% CI = 0.79–1.17, *p* = 0.68, I^2^ = 0%). It was also determined that the allele genetic model and the heterozygous and recessive genetic models had no associations with the risk of CAD.

### 3.5. Sensitivity Analyses and Publication Bias

A single study included in the meta-analysis was excluded following sequential omission. The corresponding pooled ORs were not materially altered in any subjects with either the HindIII or PvuII genotypes, suggesting the stability of the meta-analysis (data not shown). To assess the publication bias of the meta-analysis, Begg’s funnel plot and Egger’s test were performed. The funnel plot shapes were symmetrical for all the polymorphisms of HindIII and PvuII genotypes (data not shown). The statistical results did not suggest any evidence of publication bias among these studies. However, for Ser447X genotype, it was found that when the study by Ahmadi and colleagues was deleted, the sensitivity analysis was unstable and publication bias existed [22]. Therefore, it was considered that the association between Ser447X polymorphism and CAD risk still requires further research.

## 4. Discussion

In this study, we first performed a meta-analysis for the association between LPL polymorphism and CAD risk. It was found that the LPL HindIII polymorphism was positively correlated with CAD risk. In contrast, the LPL PvuII polymorphism had no association with CAD risk. Further research on the association between LPL Ser447X polymorphism and CAD risk is still needed.

The LPL gene spans over 30 kb, comprising 10 exons and nine introns on chromosome 8p22. Its cDNA is translated to a 475 amino acid proteins, including a 27 amino acid signal peptide. Several sequence variations, including BamHI, PvuII, HindIII, BstNI and Ser447X sites, have been identified by restriction fragment length polymorphisms (RFLPs) in the LPL gene [31,32,33]. Among these variations, the HindIII, Ser447X and PvuII polymorphisms were the most common and may be associated with profound alterations in plasma lipids. Recently, some studies have reported that the HindIII, Ser447X and PvuII gene plolymorphisms decreased plasma LPL activity. Furthermore, decreased plasma LPL activity was associated with elevated TG and low HDL-C levels in patient samples, which can contribute to CAD risk [34,35,36,37]. The HindIII polymorphism is located in intron 8, 495 bp from the splice-donor site, and it can affect RNA splicing [38]. The H-allele of the HindIII polymorphism could cause either enhanced enzyme activity or more efficient lipid binding [34]. The Ser447X polymorphism is located in intron 9, where cytosine (C) is replaced by guanine (G), at position 1959. This polymorphism leads to the suppression of the final two amino acids, serine and glycine at position 447 of protein [35,36]. The PvuII polymorphism is located on intron 6, 1.57 kb from the SA site. The region containing the PvuII site resembles the splicing site in its homology to the consensus sequence required for 39-splicing and the formation of the lariat structure, suggesting that C497-T change may interfere with the correct splicing of messenger RNA [17,37]. 

The association between the LPL polymorphism and CAD has been researched for thirty years. However, the results to date have been inconsistent. Currently, there are no large scale case control studies for LPL polymorphism and CAD risk. Thus, we performed a meta-analysis to study the association between LPL polymorphism and CAD risk. To analyze the association between the HindIII polymorphism and CAD risk, we reviewed seven studies including 1853 cases and 1171 controls that were conducted from 2000 to 2015. As shown in Figure 2 and Figure 3, the analysis revealed that the HindIII H^+^H^+^ genotype and the H^+^ allele genotype were significantly associated with the risk of CAD. These results were consistent with previous reports that HindIII is the most common polymorphism of LPL associated with CAD risk. However, for the Ser447X polymorphism, the association with CAD was only found in the XX genotype; the other genotype had no significant association. The difference in the XX genotype may have been caused by publication bias because two studies reported no events in the XX genotype [15,25]. More studies are needed to confirm this result. For the PvuII polymorphism, no significant association to CAD risk was detected. 

However, there are some limitations in this study. First, based on our inclusion and exclusion criteria, the available studies that could be included in this meta-analysis were moderate. Thus, the results may be influenced by factors such as random error. Second, the results were based on individual unadjusted effect estimates, whereas a more precise evaluation would be adjusted by other potential risk factors, including age, sex, drinking status, cigarette consumption, etc. Third, the lack of individual-level data prevented further analyses to identify interactions between the genetic variations and the metabolic traits.

## 5. Conclusions

In conclusion, we found that the LPL polymorphisms HindIII H^+^H^+^ genotype and H^+^ allele genotype were significantly associated with the risk of CAD. The Ser447X XX genotype was also significantly associated with the risk of CAD. However, more studies are needed to confirm these findings. In contrast, the PvuII polymorphism had no association with the risk of CAD. We have concluded that LPL HindIII polymorphism might serve as a potential biomarker for CAD risk.

## Figures and Tables

**Table 1:** Characteristics of studies included in meta-analysis.

Study	Years	Ethnicity	No. of Case/Control	Matching Criteria	Control Source	Genotyping	HWE
**HindⅢ Polymorphism**							
Abu-Amero [14]	2003	Saudi Arabian	352/410	Sex, Age	HB	PCR	Yes
Ahmadi [22]	2015	Iranian	108/89	Sex, Age	HB	PCR	Yes
Al-Jafari [15]	2012	Saudi Arabian	120/65	Sex, Age	HB	PCR	Yes
Daoud [25]	2013	Saudi Arabian	226/103	Sex, Age	HB	PCR	Yes
Pasalic [28]	2006	Croatian	132/98	Sex, Age	HB	PCR	Yes
Tanguturi [13]	2013	Indian	202/210	Sex, Age	HB	PCR	Yes
Whiting [30]	2004	American	713/196	Sex, Age	HB	PCR	Yes
**PvuII Polymorphism**							
Abu-Amero [14]	2003	Saudi Arabian	431/511	Sex, Age	HB	PCR	Yes
Al-Jafari [15]	2012	Saudi Arabian	120/65	Sex, Age	HB	PCR	Yes
Daoud [25]	2013	Saudi Arabian	226/103	Sex, Age	HB	PCR	Yes
Duman [17]	2004	Turkish	78/49	Sex, Age	HB	PCR	Yes
Georgiev [16]	2008	Macedonian	109/32	Sex, Age	HB	PCR	Yes
Isbir [27]	2003	Turkish	100/72	Sex, Age	HB	PCR	Yes
**Ser447X Polymorphism**							
Ahmadi [22]	2015	Iranian	115/89	Sex, Age	HB	PCR	Yes
Al-Jafari [15]	2012	Saudi Arabian	120/65	Sex, Age	HB	PCR	Yes
Almeida [23]	2006	Brazil	313/150	Sex, Age	HB	PCR	Yes
Aydogan [24]	2009	Turkey	41/23	Sex, Age	HB	PCR	Yes
Daoud [25]	2013	Saudi Arabian	226/103	Sex, Age	HB	PCR	Yes
Ferencak [26]	2003	Croatian	479/200	Sex, Age	HB	PCR	Yes
Pasalic [28]	2006	Croatian	132/98	Sex, Age	HB	PCR	Yes
Sawano [29]	2001	Japanese	93/96	Sex, Age	HB	PCR	Yes

HB: hospital based, HWE: Hardy–Weinberg equilibrium.

**Table 2:** Genotypes and allele frequencies of Lipoprotein Lipase (LPL) genes in patients and controls.

Author	Year	Case					Control					Sample Size	HWE (P)
**HindⅢ Polymorphism**		**H^+^H^+^**	**H^+^H^−^**	**H^−^H^−^**	**H^+^**	**H^−^**	**H^+^H^+^**	**H^+^H^−^**	**H^−^H^−^**	**H^+^**	**H^−^**		
Abu-Amero [14]	2003	189	138	25	516	188	206	173	31	585	235	352/410	Yes
Pasalic [28]	2006	78	46	8	202	62	47	45	6	139	57	132/98	Yes
Whiting [30]	2005	385	269	59	1039	387	103	77	16	283	109	713/196	Yes
Daoud [25]	2013	102	81	43	285	167	42	35	26	119	87	226/103	Yes
Al-Jafari [15]	2012	61	53	6	175	65	29	23	13	81	49	120/65	Yes
Tanguturi [13]	2013	98	72	32	268	136	70	68	72	208	212	202/210	Yes
Ahmadi [22]	2015	61	41	6	163	53	53	33	3	139	39	108/89	Yes
**Ser447X Polymorphism**		**SS**	**SX**	**XX**	**S**	**X**	**SS**	**SX**	**XX**	**S**	**X**		
Ahmadi [22]	2015	58	23	34	139	91	75	7	7	157	21	115/89	Yes
Al-Jafari [15]	2012	100	20	0	220	20	57	8	0	122	8	120/65	Yes
Almeida [23]	2007	257	47	9	561	65	115	34	1	264	36	313/150	Yes
Aydogan [24]	2009	27	10	4	64	18	17	2	4	36	10	41/23	Yes
Daoud [25]	2013	185	41	0	411	41	92	11	0	195	11	226/103	Yes
Ferencak [26]	2003	378	97	4	853	105	167	32	1	366	34	479/200	Yes
Pasalic [28]	2006	113	19	0	245	19	69	28	1	166	30	132/98	Yes
Sawano [29]	2001	82	10	1	174	12	71	23	2	145	27	93/96	Yes
**PvuII Polymorphism**		**P^+^P^+^**	**P^+^P^−^**	**P^−^P^−^**	**P^+^**	**P^−^**	**P^+^P^+^**	**P^+^P^−^**	**P^−^P^−^**	**P^+^**	**P^−^**		
Abu-Amero [14]	2003	138	225	68	501	361	182	248	81	612	410	431/511	Yes
Al-Jafari [15]	2012	50	52	18	152	88	25	28	12	78	52	120/65	Yes
Daoud [25]	2013	89	102	35	280	172	46	44	13	136	70	226/103	Yes
Duman [17]	2004	25	39	14	89	67	14	16	19	44	54	78/49	Yes
Georgiev [16]	2008	25	58	26	158	110	5	20	7	30	34	109/32	Yes
Isbir [27]	2003	37	49	14	123	77	20	40	12	80	64	100/72	Yes
